# Mummification in Ancient Egypt

**Published:** 1990-12

**Authors:** Joseph Sluglett

**Affiliations:** General Practitioner (Retd.)


### Mummification in Ancient Egypt

**Joseph Sluglett, OBE, MD**
General Practitioner (Retd.)

In the popular mind, the practice of mummification is usually associated solely with ancient Egypt, yet there is a good deal of evidence[1] that it has also been customary in other parts of the world as far away as Peru and the Canary Islands. It is well, at the outset to define quite clearly what is meant by the term, mummy. The shorter Oxford dictionary has, as its primary definition, an embalmed body, but secondarily, it also includes a human or animal body dessicated by exposure to the sun and air. It is less confusing to restrict the use of the term to a body which has been subjected to the art of the embalmers as distinct from one which has become mummified by burial in conditions such as in the hot dry sand of Egypt. In passing, it is interesting to note[2] that bodies preserved and mummified in this way by nature are, on the whole in far better condition than those of ancient royalty which had been embalmed during the times when the art was at its highest peak of excellence, that is, during the XXIst dynasty, 1085–945 BC.

The word mummy is derived from the Persian, mummia, meaning bitumen which the Persians discovered in certain locations as natural deposits and used as a sort of panacea in the belief that it possessed magical and medicinal properties. When mummies were first discovered, it was noticed that bitumen exuded from them and this led to the belief that its curative and magical powers were enhanced by contact with the bodies which the bitumen had helped to preserve. When Egyptology became more scientific, it was realised that bitumen was rarely used in ancient Egypt, not until much later in its history, but the name stuck and has remained in popular usage. The Egyptians would never have used the word mummy, it has been suggested[3] that their word would have been Sahu, which means, an embalmed cadaver.

Long after mummification was abandoned, extracts of mummies were used for all sorts of purposes. The Arab physician Avicenna who died in 1039, included its use in his Canon of Medicine, it was used by the Crusaders for the healing of wounds and in various parts of Europe for some internal conditions until the 18th century when all reference to it as a medicament disappears. Over the preceeding centuries it had been used as a condition powder for falcons, fish bait and as a dye; Othello refers to the fateful handkerchief being 'dyed in mummy.'[4] As Sir Thomas Browne, author and physician, 1605–82, so eloquently observed in Religio Medici, "Mummy is become merchandise, Mizraim cures wounds and Pharaoh is sold for balsams".

Probably the most bizzare use of mummies in fairly recent times is described in Dard Hunter's book, Papermaking, published by A. Knopf 1943. On p. 382–383, he relates how, during the American civil war, Augustus Stanwood was very concerned about the shortage of raw materials for his paper mill. He overcame this difficulty by importing several shiploads of Egyptian mummies and stripped them of their cloth wrappings and papyrus fillings which he then used to manufacture a rather coarse brown wrapping paper. When he died on March 6th 1914, the newspaper obituary notices mentioned this fact and the ensuing correspondence indicated that Stanwood was not the first and only papermaker to use this unusual material. Hunter also quotes Augustus Stanwood's son, a retired professor of international law as saying that the only competition his fatherhad to contend with in buying the mummies was the Egyptian railroad, for, during a ten year period this was the only fuel used by the Egyptian locomotives and, at that time, the supply of mummies was thought to be unlimited.

It is part of the human condition and philosophy that man has, from the earliest times been unwilling to face the prospect of complete extinction with death and the ancient Egyptians were among the earliest societies to record their belief in an after life. Indeed, they went to the most extraordinary lengths to ensure the physical survival of the body after death and this is how the practice of mummification arose. At what period in their history this started is not known but it is thought to have been about the time of the IInd dynasty or archaic period, about 3000 B.C. and it persisted until well into the Christian era, finally disappearing with the rise of Islam.

Man is the only animal to bury his dead and in remote times in Egypt the body was simply placed naked or wrapped in a sheet, into a shallow grave. The hot dry sand preserved and mummified the body and this fact was noted by the Egyptians and may have given rise to the belief in, or strengthened their hopes of, ultimate survival. Their earliest efforts cannot have been particularly successful as few of them have survived but over the centuries their technique improved reaching its best for about six hundred years, from the XVIIIth to the XXIst dynasty, 1567–945 B.C., after which it began to decline. At first it was the sole perogative of royalty but, in the course of time the privilege was extended to the nobility and by the Roman era, that is, about 100 B.C. it was available to anyone who could afford it. Despite this fairly long history there is no reference in ancient Egyptian literature to the actual details of mummification and when one considers how often Egypt had been exposed to external invasion it is surprising that there is hardly any reference to be found in contemporary literature. The last verse in Genesis records the death of Joseph, "being an hundred and ten years old; they embalmed him and he was put in a coffin in Egypt."[5] There is a meagre reference to the embalming of Asa,[6] but, surprisingly, in view of the long captivity of the Israelites in Egypt, no further mention of mummification is to be found in Exodus or anywhere else in the Old Testament. In the New Testament,[7] Lazarus is described as coming forth from his tomb, his hands and feet swathed in bandages, his face wrapped in a cloth and later on, Joseph of Arimathea embalmed the body of Jesus in myrrh and aloes before wrapping it up with the spices in "strips of linen according to Jewish burial customs".

The most important authority to whom all writers on mummification refer and usually quote almost verbatim is the Greek historian, Herodotus[9] who lived from about 484 until 424 BC. There is also a short account by Diodorus Siculus who flourished some 400 years later under Caesar and Augustus. As historians, neither is very highly regarded but they are in general agreement as to the methods employed in mummification. It must be realised, however, that as this was a religious ceremony, they would not have been allowed to witness any of it so that all their information would be based on hearsay. Further, at the times in which they wrote, the art of mummification was already in decline and not very highly thought of. A good deal of the information we now possess is derived from reading the hieroglyphs on the walls of the tombs and from the examination of the mummies themselves.

Although the methods varied from time to time over the centuries and carried on from father to son by oral tradition, with a meticulous people like the Egyptians there may well have been some written manuals of instructions which like the Dead Sea Scrolls are lying somewhere, awaiting discovery.

It would appear that there were three different methods, based mostly on the status of the deceased and how much the family could afford. The first was the most expensive and entailed the removal of the brain through the nostrils and the viscera through an incision in the left flank; it was part of their religious beliefs that life entered the body on the right side and departed on the left. Usually but not always, the heart and kidneys were left in situ. Next, the body was treated with spices and embalmed after which, it was subjected to the dessication process now thought to have been by its immersion in dry natron or simply buried in the hot dry sand. After this was completed, the body was bandaged and then put into its human shaped wooden case or series of cases, often decorated in most elaborate designs and hieroglyphs. In the second type, there was no attempt at removal of the viscera which were simply dissolved by the injection of oil of cedar via the anus after which the body was immersed in the natron. In the third method, the intestines were cleared by means of a purge and the body then immersed in the natron.

There is no doubt that, in ancient Egypt, mummification was a highly organised and flourishing industry, very much like its modern counterpart in the United States and, despite the tremendous religious ritual associated with death, there must have been some sceptics or commercially minded people like those satirised by Evelyn Waugh in "The Loved Ones". It was, after all really big business, we don't yet know how long it took to dry out the body but the whole elaborate and expensive process took seventy days, and each stage would have its appropriate hymns and prayers all the way through. Then, as now, the relatives of the deceased would come to the funeral parlour to inspect and choose from the different types of coffin or sarcophagi which were kept in stock. Many of those which have been discovered are very similar in design and have the same hieroglyphs and other decorations. All this would require quite a large staff of craftsmen and temple officials of varying degree. Similarly, the actual obsequesies would vary with the importance of the individual, royalty or nobility would have the equivalent of the Archbishop of Canterbury or York, whereas the ordinary citizen would have to be content with the local parish priest or temple official.

The treatment of the body after the drying out process had been completed varied a good deal over hundreds of years. The main purpose remained the same throughout and that was to maintain the body as far as possible in the likeness of its lifetime condition. They believed implicitly in the physical resurrection of the dead. As St. Augustine, in his sermon, "De Resurrectione Mortuorum", remarks, "I suppose then that the only people who believe in the resurrection are the Egyptians who carefully preserve their dead". They believed that, at death the Ka or soul left the body and had to wander in limbo for 3000 years before returning and it had to be given every assistance towards recognising its former human habitation. This was the reason for the most extraordinary efforts which were made to restore the body to its original state. The abdominal cavity was packed with substances of various kinds all of which had religious significance, such as linen and mud from the Nile, the source of life. Attempts were made to restore the outline of the limbs, trunk and face by subcutaneous packings through small incisions, artificial eyes of precious or semi precious stones were inserted into the sockets and make up was applied to the face. In ancient Egypt both sexes used make up; in the Bristol museum there is the mummified head of a young man with henna dyed hair still attached and traces of gold paint on his face. In the female, the eyes, breasts and mons veneris were often gilded, sometimes the whole body was so treated and occasionally this privilege was extended to commoners.

The treatment of the viscera varied with the different dynasties. At first they were all left in situ but it was soon discovered that this tended to hasten decomposition so they were removed. In earlier times they were sealed in canopic jars, four in number, each one representing of the children of Horus. Originally, all had stoppers carved in the shape of human heads but later, after the XVIIIth dynasty only one was human headed, Imsety, which held the liver. The jar with an ape head contained the lungs dedicated to Hapy, the guardian of the stomach was Duamutef, the jackal headed, while Qebhsenuef was protector of the intestines and had the head of a hawk.

In later dynasties the viscera were wrapped in separate packages which were then replaced in the body or between the knees. Occasionally, in still later dynasties canopic jars were used for a short period but eventually they were abandoned altogether. The body had to be correctly orientated with the feet pointing to the east so that on the day of resurrection it would greet the rising sun. The positioning of the hands was important, though this varied from time to time. They were placed alongside the body with extended palms or at the sides of the thighs or folded across the chest or extended to rest, one on each shoulder. All these had special meaning which varied with age, sex and period, the first being reserved for men while the second was for women and the third, maybe for royalty. Where the hands meet in front as though to shield the genitalia, this is thought to indicate members of the priestly class.

It will be readily appreciated that the death of anyone of consequence from royalty downwards imposed a very heavy financial burden on the state or relatives. One contributory factor towards the cost, was the large number of people involved, each with his own highly specialised function, from the man who made the first incision in the left flank, the man who drew out the entrails, then the embalmers and so on up to the different functionaires who did the actual bandaging. It is possible that here also there might have been different specialists for the face, each limb, fingers and so on; it would appear that modern restrictive practices in industry have a very long history!

In addition to the preparation of the body there were many other things to do. As they believed in an after life, it seemed logical to provide all those necessities and luxuries which had been enjoyed in life on earth, such as food, clothing, furniture, servants and a good deal of personal jewelry, always a status symbol in any society. In remote times all this was taken very literally and human beings and animals were slain at the funeral of the king so that they might accompany him and attend to his wants.

As far as the humans were concerned this may well have been voluntary as many of the court attendants and friends of the king might have been glad to keep his company on his last journey. In "Ur of the Chaldees"[11] Sir Leonard Woolley paints a very imaginative and moving picture of one such funeral service. Later on this practice was discontinued, no doubt because it was expensive, and models of humans and aminals called Ushabtis were substituted. Much more effort was expended on the decoration of the sarcophagi and the walls of the tombs and it is from these sources that most of our knowledge of everyday life in ancient Egypt is derived.

Some attempts have been made to assess the cost in terms of modern currency but this is rather a futile exercise as money did not have the same meaning to the Egyptians as it has for us today. An article in the Lancet in 1836 suggests[12] a figure of £225 for the most expensive mummification and £75 for the second method so that, taking into account the different values in money over the past 150 years or so, this would be a very expensive business. Nor does it end there, there is still the tomb and the cost of the funeral service to be paid for. The treasures found in the tomb of Tutankhamen give some idea of the wealth expended and it must be emphasised that he was a very minor Pharoah indeed. Who knows what treasures future excavations may reveal?

There are collections of mummies in museums all over the world and, although much has been learnt from them, a good deal of research still remains to be done. Happily, this is now being conducted on highly scientific lines which do not necessarily involve destruction of the mummy as was the case formerly. Now we use X-rays, EMI scanners, carbon 14 dating, tissue typing and blood grouping and no doubt new methods will be developed in the future.

Interest in mummies was given a tremendous boost by the excavations of Belzoni[13] in the early years of the 19th century and great public enthusiasm was shown for his exhibition in the Egyptian Hall in Piccadilly in the spring of 1821. One of the highlights was his dissection of a mummy in the summer of that year before a distinguished audience which included Royalty and a bishop as well as a large number of doctors. The Times of May 1st 1821 advertises this event but I have been unable to trace any account of the proceedings in subsequent issues of the paper. This event may well have started the fashion of visitors to Egypt bringing back mummies of which there must have been a large number offered freely for sale. Mr Salt, 1780–1827, who at one time employed Belzoni, had quite a large collection and some of them came up for sale at Sotheby's in 1835. Some were bought by the Royal College of Surgeons but others were bought privately and kept at home as antiques. Many dissections were performed during the century, not often inspired by scientific interest but more in the nature of an entertainment. The Bristol Mirror of April 1834 refers to one carried out in 1823 but the account cannot be found. The same paper, however, carries a fairly detailed report of a mummy dissection by a noted Bristol physician Dr. J. C. Pritchard at the Bristol Institution on March 31st 1834. As the bandages were unwrapped, they were found to be of good linen, often with selvage, varying from five to seven inches in width and of a total length of over six hundred yards. The sex was said to be female and it was decided that it had been mummified using the first method described by Herodotus which implied that she had been a woman of some importance. A copy of the report has been lodged with the museum, but the mummy itself has disappeared.

Dr. Thomas Pettigrew at one time Professor of Anatomy at Charing Cross Hospital, performed many of these demonstrations and seems to have made quite a living out of them. One of these[14] was on "Horiesi, the son of Nasphihiniegori, an incense bearing priest in the temple of Amon . . . from Thebes" and is described in detail by Warren Dawson in a paper entitled "Pettigrew's Demonstrations upon Mummies".[15] An intriguing note in the Lancet[16], describes "a bulbous root found in the hand of an Egyptian mummy 2000 years old, germinated on exposure and, when put in the ground grew with readiness and vigour." Alas, it doesn't say what it was that grew and modern experiments in this direction have been singularly unfruitful. There are several other references in the Lancet and one in 1836[17], was thought to have been of a mummy contemporary with Moses. In Bristol, we have a copy of the pamphlet in 1833 by Dr. John Davidson, "Embalming and Unrolling of a Mummy", published by James Ridgeway of Piccadilly and there may well be many other reports buried away in libraries and institutions.

To come to modern times, 1906 was notable for a demonstration by Margaret Murray in Manchester and in the same city in 1975, Dr. Rosalie David[18] dissected a mummy and the whole proceedings were watched by thousands on television.

Although so much interest these days seems to be concentrated on tombs, pyramids and mummies, it must not be thought that the ancient Egyptians were eternally preoccupied with death and the hereafter. On the contrary, from the evidence available, they seem to have enjoyed life to the full. Despite their high infant mortality and fairly short life expectancy and recurring wars between the north and south not to mention the numerous invasions from without, there was always the sunshine and the mystery and magic of the Nile. Life was simple and uncomplicated and their religion taught them that, at death the Pharoah would be admitted into the pantheon of their gods and that in turn, this fate would be shared by all his subjects. They saw nothing incongruous in active participation in the building of his tomb and organising his funeral during his lifetime because they too were directly involved. As Dr. Davidson, who in his time was a noted Egyptologist, remarks in the pamphlet already referred to, "The ancient Egyptians seem to have paid more attention to their sepulchres than their homes which they called Inns, because of the short period they occupied them as distinct from their graves which was forever."

### REFERENCES

1. Egyptian Mummies, Elliot and Dawson, 1924.
2. Ibidem.
3. Radiological Examination of the Egyptian Mummies in the Archaeological Museum of Madrid, 1978.
4. Othello, Act II, Scene IV.
5. The Old Testament, The New English Bible.
6. Ibidem, 11 Chronicles, 16, 14.
7. The New Testament, The New English Bible, John, **11**, 44.
8. Ibidem. John 39–40.
9. Herodotus, The Histories, Aubrey de Selincourt, The Penguin Classics.
10. Augustine Sanctus Aurelius. Sermo CCCLXI.
11. Ur of the Chaldees, Sir Leonard Woolley, Pelican Books, 1942.
12. The Lancet, 1836, Vol. 30, p. 367.
13. The Great Belzoni, Stanley Hayes, Putnam, 1959.
14. The Lancet 1833, Vol. 25, pps. 646, 693.
15. Journal of Egyptian Archaeology, Vol. 20, pps. 170–182.
16. The Lancet, 1829, Vol 11, p. 432.
17. The Lancet, 1836, Vol. 30, p. 367.
18. Mysteries of the Mummies, Rosalie David, Book Club Associates, 1978.

### BIBLIOGRAPHY

The Egyptian Kingdoms, Rosalie David, Phaidon Press, 1975.

Egyptian Mythology, Paul Hamlyn Ltd. 1965.

Tutankhamen, Christiane Desroches-Noblecourt, Penguin Books, 1972.

The Spirit of Ancient Egypt, W. A. Ward, Khayats, Beirut,

